# 3-(4-Bromo­phen­yl)-4-[2-(4-nitro­phen­yl)hydrazin­yl]furan-2(5*H*)-one

**DOI:** 10.1107/S1600536811043947

**Published:** 2011-10-29

**Authors:** Zhu-Ping Xiao, Li-Cheng Yi, Jia-Liang Li, Bo Zhang, Mei-Ling Liao

**Affiliations:** a College of Chemistry and Chemical Engineering, Jishou University, Jishou 416000, People’s Republic of China

## Abstract

In the title compound, C_16_H_12_BrN_3_O_4_, the furan-2(5*H*)-one ring forms a dihedral angle of 33.19 (9)° with the 4-bromo­benzene unit and is nearly perpendicular to the 4-nitro­benzene segment, making a dihedral angle of 89.93 (10)°. In the crystal, N—H⋯O hydrogen bonds link the mol­ecules, generating an infinite chain along [010]. The chains are linked into a three-dimensional network by C—H⋯O, C—H⋯π and π–π contacts [centroid–centroid separation = 3.805 (2) Å].

## Related literature

For background to 3-aryl­furan-2(5*H*)-ones as anti­bacterial agents, see: Xiao *et al.* (2011*a* ▶,**b* ▶,c* ▶). For further details of C—H⋯π inter­actions, see: Castillo *et al.* (2009 ▶); Li *et al.* (2007 ▶); Trilleras *et al.* (2009 ▶).
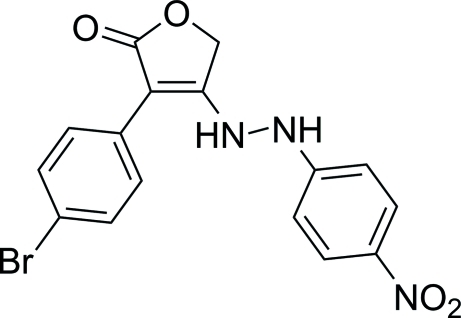


## Experimental

### 

#### Crystal data

C_16_H_12_BrN_3_O_4_
*M* *_r_* = 390.20
Orthorhombic, 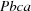

*a* = 14.4725 (11) Å
*b* = 6.7744 (5) Å
*c* = 31.310 (2) Å
*V* = 3069.8 (4) Å^3^
*Z* = 8
Mo *K*α radiation
μ = 2.71 mm^−1^
*T* = 296 K
0.30 × 0.20 × 0.20 mm

#### Data collection

Bruker SMART APEX CCD diffractometer
Absorption correction: multi-scan (*SADABS*; Sheldrick, 1996 ▶) *T*_min_ = 0.498, *T*_max_ = 0.614
16108 measured reflections
3022 independent reflections
2039 reflections with *I* > 2σ(*I*)
*R*_int_ = 0.046

#### Refinement

*R*[*F* ^2^ > 2σ(*F* ^2^)] = 0.036
*wR*(*F* ^2^) = 0.096
*S* = 1.02
3022 reflections
224 parameters
H atoms treated by a mixture of independent and constrained refinement
Δρ_max_ = 0.58 e Å^−3^
Δρ_min_ = −0.63 e Å^−3^

### 

Data collection: *SMART* (Bruker, 2007 ▶); cell refinement: *SAINT* (Bruker, 2007 ▶); data reduction: *SAINT*; program(s) used to solve structure: *SHELXS97* (Sheldrick, 2008 ▶); program(s) used to refine structure: *SHELXL97* (Sheldrick, 2008 ▶); molecular graphics: *SHELXTL* (Sheldrick, 2008 ▶); software used to prepare material for publication: *SHELXL97*.

## Supplementary Material

Crystal structure: contains datablock(s) global, I. DOI: 10.1107/S1600536811043947/hb6467sup1.cif

Structure factors: contains datablock(s) I. DOI: 10.1107/S1600536811043947/hb6467Isup2.hkl

Supplementary material file. DOI: 10.1107/S1600536811043947/hb6467Isup3.cml

Additional supplementary materials:  crystallographic information; 3D view; checkCIF report

## Figures and Tables

**Table 1:** Hydrogen-bond geometry (Å, °) *Cg*3 is the centroid of the C1–C6 ring.

*D*—H⋯*A*	*D*—H	H⋯*A*	*D*⋯*A*	*D*—H⋯*A*
N1—H1*A*⋯O1^i^	0.80 (4)	2.13 (4)	2.913 (3)	163 (4)
N2—H2*A*⋯O2^i^	0.91 (3)	2.50 (3)	2.979 (3)	113 (2)
C9—H9*B*⋯O3^ii^	0.97	2.45	3.380 (4)	161
C2—H2⋯*Cg*3^iii^	0.93	2.86	3.676 (3)	147

Symmetry codes: (i) 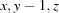; (ii) 
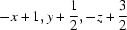
; (iii) 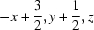.

**Fig. 1.**
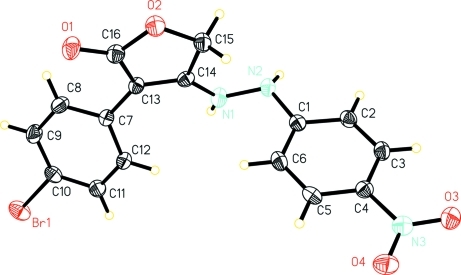
Molecular structure of the title compound, showing displacement ellipsoids drawn at the 30% probability level.

**Fig. 2.**
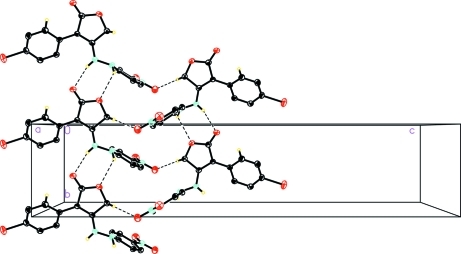
A dimeric line is formed through intermolecular N—H···O and C—H···O hydrogen bonds. For the sake of clarity, the H atoms have been omitted except that involving in hydrogen bonds.

**Fig. 3.**
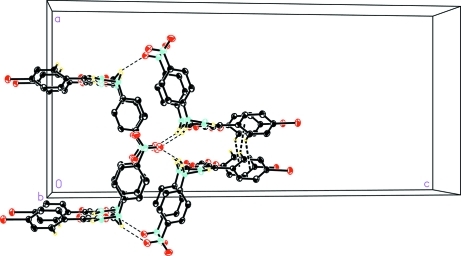
A two-dimensional layer is generated through intermolecular C—H···π contacts. For the sake of clarity, the H atoms have been omitted except that involving in hydrogen bonds.

**Fig. 4.**
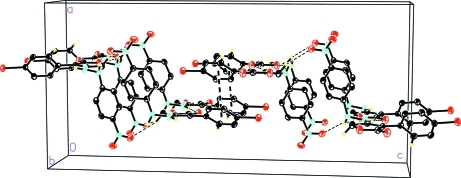
A three-dimensional network is finally formed through π–π interactions. For the sake of clarity, the H atoms have been omitted except that involving in hydrogen bonds.

## supplementary crystallographic information

### Comment

Furan-2(5*H*)-one framework is a part of many natural and synthetic compounds, which possess useful biological activities including anti-inflammatory and antitumor activity. Recently, we demostrated that 3-arylfuran-2(5*H*)-ones as antibacterial agent, some of which are potent tyrosyl t-RNA synthase inhibitors (Xiao *et al.*, 2011a, 2011b and 2011c). Herein, we reported the crystal structure of the title compound (I) (Fig. 1).

The bond length of C7—C10 is 1.350 (4) Å and was assigned as a double bond, and the title compound was therefore identified as a furan-2(5*H*)-one (scheme 1). The torsion angle of N2—N1—C10—C7 is 174.8 (3) °, indicating that N1 may adopt *sp*^2^ hybridization. Therefore the *p* orbital of N1 is conjugated with the π molecular orbital of C7—C10 double bond, which shortens the N1—C10 bond from 1.48 Å to 1.341 (4) Å. However, the torsion angle of N1—N2—C11—C16 is 21.9 (4), indicating that N2 is unlikely to have *sp*^2^ hybridization. In fact, the angle of C11—N2—H2A, C11—N2—N1 and N1—N2—H2A are 114.83 (184), 110.30 (216) and 118.11 (24), respectively. These angles are in the range of 108 to 120 °, indicating that N2 may show a type of hybridization between *sp*^2^ and *sp*^3^. This resulted in a decrease of the overlap between the nitrogen (N2) and the benzene π-orbital and elongates the N2—C11 bond (1.393 (4) Å) in comparsion with N1—C10.

In the extended structure of I, a line of molecules is generated along the *b* axis through N1—H1A···O1 and N2—H2A···O2 hydrogen bonds characterized by a graph-set motif of *R*_2_^2^(7) (Fig. 2). Utilizing the oxygen in the nitro group as acceptor, C—H···O hydrogen bonds link pairs of the resulted lines into centrosymmetric dimers, which are generated by edge fused graph-set motifs of *R*_3_^3^(23) (Fig.2).

Adjacent dimeric lines are linked together *via* C2—H2···π contacts, forming an infinite two-dimensional layer parallel to the plane (001). The H···π length of the typical C—H···π hydrogen bond is in the range of 2.70 to 3.10 Å (Trilleras *et al.*, 2009; Castillo, *et al.*, 2009; Li, *et al.*, 2007). C2—H2···π in compound I is thus considerated as a moderate contact with H···*Cg* length of 2.86 Å, where *Cg* is the centroid of C1 to C6 (Fig. 3). The resulted layers lie parallel to the plane (001), which are further linked to form its final three-dimensional network through π–π interactions with center-center length of 3.805 (2) Å (Fig. 4).

### Experimental

3-(4-Bromophenyl)-4-hydroxyfuran-2(5*H*)-one (0.51 g, 2 mmol) was added to a mixture of 4-nitrophenylhydrazine (0.37 g, 2.4 mmol) and *p*-toluene sulphonic acid (13.6 mg, 0.08 mmol). The resulted mixture was heated to 375 K for 30 min. Fifteen ml of toluene was then added and refluxed for 7 h. After toluene was removed under reduced pressure, the residue was purified by column chromatography on silica gel, eluting with EtOAc/petroleum ether (*v*/*v* = 2/1), which was partially evaporated to give colorless blocks of (I).

### Refinement

The H atoms bonded to N1 and N2 were located in difference Fourier maps, and all other H atoms were placed in geometrically idealized positions and constrained to ride on their parent atoms with C—H = 0.93 Å for aromatic H atoms and 0.97 Å for CH_2_ type H atoms, respectively. *U*_iso_(H) values were set at 1.2 times *U*_eq_(C) for both aromatic C and the CH_2_ group.

### Crystal data

**Table:**

C_16_H_12_BrN_3_O_4_	*F*(000) = 1568
*M**_r_* = 390.20	*D*_x_ = 1.689 Mg m^−^^3^
Orthorhombic, *P**b**c**a*	Mo *K*α radiation, λ = 0.71073 Å
Hall symbol: -P 2ac 2ab	Cell parameters from 1957 reflections
*a* = 14.4725 (11) Å	θ = 2.3–24.6°
*b* = 6.7744 (5) Å	µ = 2.71 mm^−^^1^
*c* = 31.310 (2) Å	*T* = 296 K
*V* = 3069.8 (4) Å^3^	Block, colorless
*Z* = 8	0.30 × 0.20 × 0.20 mm

### Data collection

**Table:**

Bruker SMART APEX CCD diffractometer	3022 independent reflections
Radiation source: fine-focus sealed tube	2039 reflections with *I* > 2σ(*I*)
graphite	*R*_int_ = 0.046
φ and ω scan	θ_max_ = 26.0°, θ_min_ = 1.9°
Absorption correction: multi-scan (*SADABS*; Sheldrick, 1996)	*h* = −17→17
*T*_min_ = 0.498, *T*_max_ = 0.614	*k* = −8→7
16108 measured reflections	*l* = −38→35

### Refinement

**Table:**

Refinement on *F*^2^	Primary atom site location: structure-invariant direct methods
Least-squares matrix: full	Secondary atom site location: difference Fourier map
*R*[*F*^2^ > 2σ(*F*^2^)] = 0.036	Hydrogen site location: inferred from neighbouring sites
*wR*(*F*^2^) = 0.096	H atoms treated by a mixture of independent and constrained refinement
*S* = 1.02	*w* = 1/[σ^2^(*F*_o_^2^) + (0.0432*P*)^2^ + 1.6464*P*] where *P* = (*F*_o_^2^ + 2*F*_c_^2^)/3
3022 reflections	(Δ/σ)_max_ = 0.001
224 parameters	Δρ_max_ = 0.58 e Å^−^^3^
0 restraints	Δρ_min_ = −0.63 e Å^−^^3^

### Special details

**Table:**

Geometry. All e.s.d.'s (except the e.s.d. in the dihedral angle between two l.s. planes) are estimated using the full covariance matrix. The cell e.s.d.'s are taken into account individually in the estimation of e.s.d.'s in distances, angles and torsion angles; correlations between e.s.d.'s in cell parameters are only used when they are defined by crystal symmetry. An approximate (isotropic) treatment of cell e.s.d.'s is used for estimating e.s.d.'s involving l.s. planes.
Refinement. Refinement of *F*^2^ against ALL reflections. The weighted *R*-factor *wR* and goodness of fit *S* are based on *F*^2^, conventional *R*-factors *R* are based on *F*, with *F* set to zero for negative *F*^2^. The threshold expression of *F*^2^ > σ(*F*^2^) is used only for calculating *R*-factors(gt) *etc*. and is not relevant to the choice of reflections for refinement. *R*-factors based on *F*^2^ are statistically about twice as large as those based on *F*, and *R*- factors based on ALL data will be even larger.

### Fractional atomic coordinates and isotropic or equivalent isotropic displacement parameters (Å^2^)

**Table:**

	*x*	*y*	*z*	*U*_iso_*/*U*_eq_	
Br1	0.61747 (3)	0.27111 (6)	0.394041 (10)	0.05931 (16)	
C1	0.62850 (19)	0.4835 (4)	0.53598 (9)	0.0311 (6)	
C2	0.6776 (2)	0.5813 (5)	0.50422 (10)	0.0429 (8)	
H2	0.7131	0.6909	0.5114	0.051*	
C3	0.6746 (2)	0.5187 (5)	0.46235 (10)	0.0452 (8)	
H3A	0.7078	0.5851	0.4414	0.054*	
C4	0.6220 (2)	0.3580 (5)	0.45199 (9)	0.0390 (7)	
C5	0.5723 (2)	0.2560 (4)	0.48239 (9)	0.0407 (7)	
H5	0.5371	0.1465	0.4749	0.049*	
C6	0.5761 (2)	0.3203 (4)	0.52416 (9)	0.0361 (7)	
H6	0.5427	0.2529	0.5449	0.043*	
C7	0.63070 (19)	0.5525 (4)	0.58069 (9)	0.0316 (6)	
C8	0.6358 (2)	0.7596 (4)	0.59231 (10)	0.0389 (7)	
C9	0.6233 (2)	0.5875 (4)	0.65466 (9)	0.0407 (7)	
H9A	0.5653	0.5791	0.6701	0.049*	
H9B	0.6736	0.5581	0.6741	0.049*	
C10	0.62402 (19)	0.4491 (4)	0.61743 (9)	0.0329 (7)	
C11	0.5150 (2)	0.1371 (4)	0.67875 (9)	0.0326 (7)	
C12	0.5037 (2)	0.0240 (4)	0.71579 (9)	0.0368 (7)	
H12	0.5552	−0.0264	0.7298	0.044*	
C13	0.4173 (2)	−0.0127 (4)	0.73158 (9)	0.0383 (7)	
H13	0.4098	−0.0871	0.7563	0.046*	
C14	0.3413 (2)	0.0617 (4)	0.71039 (9)	0.0352 (7)	
C15	0.3506 (2)	0.1675 (5)	0.67330 (9)	0.0399 (7)	
H15	0.2986	0.2133	0.6589	0.048*	
C16	0.4374 (2)	0.2055 (4)	0.65750 (9)	0.0396 (7)	
H16	0.4441	0.2774	0.6324	0.048*	
H2A	0.649 (2)	0.101 (5)	0.6741 (11)	0.057 (11)*	
N1	0.61816 (19)	0.2544 (4)	0.62466 (9)	0.0414 (6)	
N2	0.60423 (18)	0.1894 (4)	0.66655 (8)	0.0368 (6)	
N3	0.24976 (19)	0.0297 (4)	0.72818 (8)	0.0421 (6)	
O1	0.63888 (16)	0.9067 (3)	0.57072 (7)	0.0497 (6)	
O2	0.63469 (16)	0.7778 (3)	0.63613 (7)	0.0464 (6)	
O3	0.24263 (16)	−0.0574 (3)	0.76267 (7)	0.0525 (6)	
O4	0.18281 (16)	0.0904 (4)	0.70812 (8)	0.0560 (6)	
H1A	0.627 (2)	0.175 (6)	0.6061 (11)	0.056*	

### Atomic displacement parameters (Å^2^)

**Table:**

	*U*^11^	*U*^22^	*U*^33^	*U*^12^	*U*^13^	*U*^23^
Br1	0.0609 (3)	0.0811 (3)	0.0360 (2)	0.0105 (2)	−0.00079 (16)	−0.01532 (18)
C1	0.0306 (15)	0.0285 (15)	0.0342 (15)	0.0027 (13)	0.0037 (12)	0.0014 (12)
C2	0.0419 (18)	0.0435 (19)	0.0432 (18)	−0.0119 (15)	0.0059 (15)	−0.0043 (15)
C3	0.046 (2)	0.052 (2)	0.0378 (17)	−0.0066 (17)	0.0116 (15)	0.0034 (16)
C4	0.0411 (18)	0.0452 (19)	0.0308 (16)	0.0082 (15)	−0.0015 (14)	−0.0065 (14)
C5	0.0483 (19)	0.0339 (17)	0.0399 (17)	−0.0005 (15)	−0.0026 (14)	−0.0024 (15)
C6	0.0403 (18)	0.0313 (16)	0.0367 (16)	−0.0026 (14)	0.0049 (13)	0.0014 (13)
C7	0.0353 (16)	0.0270 (15)	0.0324 (15)	−0.0005 (12)	0.0044 (13)	−0.0015 (12)
C8	0.0439 (18)	0.0331 (18)	0.0398 (17)	−0.0002 (14)	0.0019 (13)	−0.0024 (15)
C9	0.056 (2)	0.0332 (17)	0.0332 (16)	−0.0017 (15)	0.0021 (14)	−0.0028 (13)
C10	0.0310 (15)	0.0302 (16)	0.0373 (16)	−0.0018 (12)	0.0035 (13)	−0.0052 (12)
C11	0.0422 (17)	0.0234 (14)	0.0321 (15)	0.0007 (13)	−0.0022 (13)	−0.0043 (12)
C12	0.0414 (18)	0.0335 (17)	0.0356 (16)	0.0032 (13)	−0.0064 (14)	0.0046 (14)
C13	0.0475 (19)	0.0344 (17)	0.0331 (16)	−0.0005 (14)	0.0004 (14)	0.0046 (13)
C14	0.0371 (17)	0.0340 (17)	0.0346 (16)	−0.0010 (13)	0.0005 (13)	−0.0043 (13)
C15	0.0419 (18)	0.0434 (18)	0.0343 (16)	0.0065 (14)	−0.0102 (14)	−0.0022 (14)
C16	0.053 (2)	0.0349 (17)	0.0310 (15)	0.0034 (15)	−0.0035 (14)	0.0033 (13)
N1	0.0598 (17)	0.0287 (15)	0.0357 (14)	−0.0025 (13)	0.0105 (13)	−0.0037 (11)
N2	0.0429 (16)	0.0332 (14)	0.0343 (14)	0.0002 (12)	0.0031 (12)	0.0036 (12)
N3	0.0442 (16)	0.0362 (15)	0.0458 (15)	0.0023 (12)	−0.0006 (14)	−0.0066 (13)
O1	0.0742 (17)	0.0283 (12)	0.0466 (13)	−0.0027 (11)	0.0062 (12)	0.0009 (10)
O2	0.0715 (16)	0.0289 (12)	0.0389 (12)	−0.0033 (11)	0.0000 (11)	−0.0082 (9)
O3	0.0493 (14)	0.0603 (15)	0.0478 (14)	−0.0014 (12)	0.0069 (11)	0.0025 (12)
O4	0.0396 (13)	0.0637 (16)	0.0648 (15)	0.0070 (12)	−0.0068 (12)	−0.0035 (13)

### Geometric parameters (Å, °)

**Table:**

Br1—C4	1.909 (3)	C9—H9B	0.9700
C1—C2	1.390 (4)	C10—N1	1.341 (4)
C1—C6	1.391 (4)	C11—C16	1.385 (4)
C1—C7	1.476 (4)	C11—N2	1.393 (4)
C2—C3	1.379 (4)	C11—C12	1.399 (4)
C2—H2	0.9300	C12—C13	1.368 (4)
C3—C4	1.368 (4)	C12—H12	0.9300
C3—H3A	0.9300	C13—C14	1.380 (4)
C4—C5	1.378 (4)	C13—H13	0.9300
C5—C6	1.380 (4)	C14—C15	1.371 (4)
C5—H5	0.9300	C14—N3	1.453 (4)
C6—H6	0.9300	C15—C16	1.374 (4)
C7—C10	1.350 (4)	C15—H15	0.9300
C7—C8	1.451 (4)	C16—H16	0.9300
C8—O1	1.205 (3)	N1—N2	1.398 (4)
C8—O2	1.378 (4)	N1—H1A	0.80 (4)
C9—O2	1.423 (4)	N2—H2A	0.91 (3)
C9—C10	1.496 (4)	N3—O4	1.226 (3)
C9—H9A	0.9700	N3—O3	1.235 (3)
			
C2—C1—C6	117.9 (3)	N1—C10—C9	119.0 (3)
C2—C1—C7	121.1 (3)	C7—C10—C9	109.8 (2)
C6—C1—C7	121.1 (3)	C16—C11—N2	122.3 (3)
C3—C2—C1	121.2 (3)	C16—C11—C12	119.1 (3)
C3—C2—H2	119.4	N2—C11—C12	118.3 (3)
C1—C2—H2	119.4	C13—C12—C11	120.4 (3)
C4—C3—C2	119.2 (3)	C13—C12—H12	119.8
C4—C3—H3A	120.4	C11—C12—H12	119.8
C2—C3—H3A	120.4	C12—C13—C14	119.2 (3)
C3—C4—C5	121.7 (3)	C12—C13—H13	120.4
C3—C4—Br1	119.3 (2)	C14—C13—H13	120.4
C5—C4—Br1	118.9 (2)	C15—C14—C13	121.3 (3)
C4—C5—C6	118.4 (3)	C15—C14—N3	119.5 (3)
C4—C5—H5	120.8	C13—C14—N3	119.2 (3)
C6—C5—H5	120.8	C14—C15—C16	119.5 (3)
C5—C6—C1	121.7 (3)	C14—C15—H15	120.2
C5—C6—H6	119.2	C16—C15—H15	120.2
C1—C6—H6	119.2	C15—C16—C11	120.4 (3)
C10—C7—C8	107.0 (3)	C15—C16—H16	119.8
C10—C7—C1	129.9 (3)	C11—C16—H16	119.8
C8—C7—C1	123.0 (3)	C10—N1—N2	118.5 (3)
O1—C8—O2	119.0 (3)	C10—N1—H1A	122 (3)
O1—C8—C7	131.4 (3)	N2—N1—H1A	119 (3)
O2—C8—C7	109.6 (3)	C11—N2—N1	118.1 (2)
O2—C9—C10	104.4 (2)	C11—N2—H2A	115 (2)
O2—C9—H9A	110.9	N1—N2—H2A	110 (2)
C10—C9—H9A	110.9	O4—N3—O3	122.8 (3)
O2—C9—H9B	110.9	O4—N3—C14	118.3 (3)
C10—C9—H9B	110.9	O3—N3—C14	118.9 (3)
H9A—C9—H9B	108.9	C8—O2—C9	109.0 (2)
N1—C10—C7	131.2 (3)		
			
C6—C1—C2—C3	−0.1 (5)	O2—C9—C10—C7	−2.7 (3)
C7—C1—C2—C3	−179.1 (3)	C16—C11—C12—C13	−2.2 (4)
C1—C2—C3—C4	0.2 (5)	N2—C11—C12—C13	172.9 (3)
C2—C3—C4—C5	−0.3 (5)	C11—C12—C13—C14	0.5 (4)
C2—C3—C4—Br1	179.9 (2)	C12—C13—C14—C15	1.6 (5)
C3—C4—C5—C6	0.3 (5)	C12—C13—C14—N3	−177.4 (3)
Br1—C4—C5—C6	−179.9 (2)	C13—C14—C15—C16	−1.9 (4)
C4—C5—C6—C1	−0.1 (5)	N3—C14—C15—C16	177.1 (3)
C2—C1—C6—C5	0.0 (4)	C14—C15—C16—C11	0.1 (4)
C7—C1—C6—C5	179.0 (3)	N2—C11—C16—C15	−173.0 (3)
C2—C1—C7—C10	−148.8 (3)	C12—C11—C16—C15	1.9 (4)
C6—C1—C7—C10	32.2 (5)	C7—C10—N1—N2	−174.8 (3)
C2—C1—C7—C8	34.9 (4)	C9—C10—N1—N2	5.0 (4)
C6—C1—C7—C8	−144.0 (3)	C16—C11—N2—N1	−21.9 (4)
C10—C7—C8—O1	−176.9 (3)	C12—C11—N2—N1	163.1 (2)
C1—C7—C8—O1	0.1 (5)	C10—N1—N2—C11	99.7 (3)
C10—C7—C8—O2	1.7 (3)	C15—C14—N3—O4	3.7 (4)
C1—C7—C8—O2	178.7 (3)	C13—C14—N3—O4	−177.3 (3)
C8—C7—C10—N1	−179.4 (3)	C15—C14—N3—O3	−176.5 (3)
C1—C7—C10—N1	3.8 (5)	C13—C14—N3—O3	2.6 (4)
C8—C7—C10—C9	0.7 (3)	O1—C8—O2—C9	175.3 (3)
C1—C7—C10—C9	−176.0 (3)	C7—C8—O2—C9	−3.5 (3)
O2—C9—C10—N1	177.4 (3)	C10—C9—O2—C8	3.7 (3)

### Hydrogen-bond geometry (Å, °)

**Table:**

Cg3 is the centroid of the C1–C6 ring.

**Table:**

*D*—H···*A*	*D*—H	H···*A*	*D*···*A*	*D*—H···*A*
N1—H1A···O1^i^	0.80 (4)	2.13 (4)	2.913 (3)	163 (4)
N2—H2A···O2^i^	0.91 (3)	2.50 (3)	2.979 (3)	113 (2)
C9—H9B···O3^ii^	0.97	2.45	3.380 (4)	161
C2—H2···Cg3^iii^	0.93	2.86	3.676 (3)	147

Symmetry codes: (i) *x*, *y*−1, *z*; (ii) −*x*+1, *y*+1/2, −*z*+3/2; (iii) −*x*+3/2, *y*+1/2, *z*.
